# Influence of Anoctamin-4 and -9 on ADAM10 and ADAM17 Sheddase Function

**DOI:** 10.3390/membranes12020123

**Published:** 2022-01-20

**Authors:** Sinje Leitzke, Jana Seidel, Björn Ahrens, Rainer Schreiber, Karl Kunzelmann, Maria Sperrhacke, Sucharit Bhakdi, Karina Reiss

**Affiliations:** 1 Department of Dermatology, University of Kiel, 24105 Kiel, Germany; sleitzke@dermatology.uni-kiel.de (S.L.); jseidel@dermatology.uni-kiel.de (J.S.); bahrens@dermatology.uni-kiel.de (B.A.); msperrhacke@dermatology.uni-kiel.de (M.S.); 2 Physiological Institute, University of Regensburg, Universitätsstraße 31, 93053 Regensburg, Germany; Rainer.Schreiber@vkl.uni-regensburg.de (R.S.); karl.kunzelmann@vkl.uni-regensburg.de (K.K.); 3 Independent Researcher, 24105 Kiel, Germany; Bhakdi_Sucharit@protonmail.com

**Keywords:** scramblases, anoctamins, phosphatidylserine, ADAM17, ADAM10, shedding

## Abstract

Ca^2+^-activated Cl^−^ channels (TMEM16, also known as anoctamins) perform important functions in cell physiology, including modulation of cell proliferation and cancer growth. Many members, including TMEM16F/ANO6, additionally act as Ca^2+^-activated phospholipid scramblases. We recently presented evidence that ANO6-dependent surface exposure of phosphatidylserine (PS) is pivotal for the disintegrin-like metalloproteases ADAM10 and ADAM17 to exert their sheddase function. Here, we compared the influence of seven ANO family members (ANO1, 4, 5, 6, 7, 9, and 10) on ADAM sheddase activity. Similar to ANO6, overexpression of ANO4 and ANO9 led to increased release of ADAM10 and ADAM17 substrates, such as betacellulin, TGFα, and amphiregulin (AREG), upon ionophore stimulation in HEK cells. Inhibitor experiments indicated that ANO4/ANO9-mediated enhancement of TGFα-cleavage broadened the spectrum of participating metalloproteinases. Annexin V-staining demonstrated increased externalisation of PS in ANO4/ANO9-overexpressing cells. Competition experiments with the soluble PS-headgroup phosphorylserine indicated that the ANO4/ANO9 effects were due to increased PS exposure. Overexpression of ANO4 or ANO9 in human cervical cancer cells (HeLa), enhanced constitutive shedding of the growth factor AREG and increased cell proliferation. We conclude that ANO4 and ANO9, by virtue of their scramblase activity, may play a role as important regulators of ADAM-dependent cellular functions.

## 1. Introduction

Disintegrin and metalloproteinases (ADAMs) 10 and 17 can cleave a multitude of membrane-bound proteins in cis to effect release of their ectodomains, a process designated as “shedding”. ADAM17 promotes inflammation through release of tumor necrosis factor α (TNFα) [1,2,3] and modulates cell function via shedding of epidermal growth factor (EGF) receptor ligands, prominent among which are amphiregulin (AREG), epiregulin, transforming growth factor-alpha (TGFα) and heparin-binding EGF (HB-EGF) [4]. ADAM10 is critically involved in Notch receptor signaling [5] and liberates a number of adhesion molecules [6,7,8], as well as the EGFR-ligands betacellulin (BTC) and epidermal growth factor (EGF) [9].

Constitutive ADAM10/17-mediated shedding can be enhanced by elevation of intracellular Ca^2+^ [10,11,12,13]. In addition, phorbol esters stimulate ADAM17 in a Ca^2+^-independent manner [14].

We have recently obtained evidence that plasma membrane dynamics play an important role in controlling sheddase function. Asymmetric distribution of phospholipids in the plasma membrane is a hallmark of the living cell. Phosphatidylcholine and sphingomyelin reside primarily in the outer, and the negatively charged phosphatidylserine (PS) primarily in the inner leaflet. Externalization of PS is promoted by elevation of intracellular Ca^2+^, as well as by phorbol esters. We discovered that PS-externalization immediately upregulates ADAM10 and ADAM17 sheddase activity, apparently by promoting the interaction of the catalytic domains of the enzymes with their substrates [15,16].

Phospholipid membrane asymmetry is upheld by ATP-dependent “flippases” that continuously shuttle anionic phospholipids to the cytosolic membrane leaflet. Scramblases are the functional counterparts of flippases. These integral membrane proteins promote vertical movement of phospholipids back along their concentration gradient [17,18]. Two families of human scramblases, the TMEM16/Anoctamin and the Xkr family, have been identified to date. The ten members of the first family are designated TMEM16A to K or Anoctamin (ANO) 1–10 [19,20,21]. ANO1 and ANO2 function as Ca^2+^-activated chloride channels and apparently lack scramblase activity, but they are listed within the family due to structural similarity. The Xkr family comprises nine members that are less well characterized than the Anoctamins. One member, namely XKR8, seems to play a major role for acceleration of PS-exposure during apoptosis [22,23].

We have recently presented evidence for a direct connection between the function of scramblases and shedding activity of ADAM10/17. This was disclosed in a series of studies that utilized ANO6 as the model scramblase. Overexpression of ANO6 always led to enhancement of sheddase activity, and this was shown to be due to increased PS-exposure effected by the scramblase [16,24].

Alterations in activity of both scramblases and ADAMs are associated with immune diseases and tumorigenesis [25,26,27,28,29]. Investigations are, thus, called for to uncover further links between the two networks.

In this communication, we report on experiments conducted with ANO1, ANO4, ANO5, ANO7, ANO9, and ANO10 in comparison with ANO6, on the sheddase function of ADAM10 and ADAM17. The results were not entirely as might be expected. Similar links were noted between the sheddases and ANO4 and ANO9, but not with ANO5, ANO7, and ANO10. Possible reasons for these divergent findings are discussed.

## 2. Materials and Methods

### 2.1. Reagents and Antibodies

Annexin V-AF568 and Hoechst 33342 were purchased from Thermo Fisher Scientific (Waltham, MA, USA). O-phospho-L-serine (OPS) and Phorbol 12-myristate-13-acetate (PMA) were obtained from Sigma Aldrich (St. Louis, MO, USA). Ionomycin (IO) was purchased from Merck Millipore (Darmstadt, Germany). Hydroxamate-based ADAM17/ADAM10 inhibitor GW280264X [30] was purchased from Aeobious (Gloucester, MA, USA). Marimastat and ADAM10 inhibitor GI254023X [31] were purchased from Tocris Bioscience (Bristol, UK). Additional antibodies used: ANO1 (Bio-Techne, Abingdon, UK), ANO4 (Aviva Systems Biology, San Diego, CA, USA), ANO5 (Abcam, Cambridge, UK), ANO6 and ANO7 (OriGene, Rockville, MD, USA), ANO9 and ANO10 (LSBio, Seattle, WA, USA), and GAPDH (Novus Biologicals, Littleton, CO, USA). Secondary antibodies for automated Western were obtained from Protein Simple (San Jose, CA, USA) or Novus Biologicals (Novus Biologicals, Littleton, CO, USA).

### 2.2. Cell Culture

HEK293T and HeLa cells were purchased from Sigma Aldrich (St. Louis, MO, USA). Cells were grown in high glucose DMEM (Thermo Fisher Scientific, Waltham, MA, USA) supplemented with 10% fetal calf serum (FCS, PAA Laboratories, Cölde, Germany) and 1% penicillin/streptomycin (Pen/Strep, PAA Laboratories, Cölde, Germany).

### 2.3. Expression Vectors and Transfection

The expression vectors for human ANO1-V5/his, ANO4-V5/his, ANO5-V5/his, ANO6-V5/his, ANO7-V5/his, ANO9-V5/his, and ANO10-V5/his were kindly provided by Karl Kunzelmann (University of Regensburg, Regensburg, Germany). The plasmids for alkaline phosphatase (AP)-tagged AREG, TGFα and BTC were kindly provided by Carl P. Blobel (Hospital for Special Surgery, New York, NY, USA). A pcDNA3.1 vector was purchased from Thermo Fisher Scientific (Waltham, MA, USA).

HEK293T and HeLa cells were transfected using TurboFect^TM^ Transfection Reagent (Thermo Fisher Scientific, Waltham, MA, USA) according to the manufacturer’s instructions. Then, 48 h after transfection of expression vectors, cell medium was replaced by fresh DMEM. Transfection efficiency was always controlled in parallel by Western blot analyses (Supplementary Figures S1 and S2). The pcDNA3.1 vector was used as a mock control.

### 2.4. AP-Substrate Shedding Assay

HEK293T cells were seeded in 12-well plates. Forty-eight hours after transfection, medium was replaced by fresh serum free DMEM. For inhibitor experiments, cells were pre-incubated with the indicated concentrations of GI254023X (GI), GW280264X (GW), marimastat (MM), or OPS for 15 min. Thereafter, cells were treated, or not treated, with ionomycin (1 µM, 30 min). Supernatants and cell lysates were collected and measured for alkaline phosphatase (AP) activity at A405 nm employing the AP substrate 4-nitrophenyl phosphate (Sigma Aldrich, St. Louis, MO, USA). Shown is the relative AP activity in the supernatant compared to the total AP activity of supernatant plus cell lysates.

### 2.5. AREG ELISA

HeLa cells were seeded in 6-well plates. Twenty-four hours after transfection, cell culture medium was replaced by fresh medium with or without marimastat (MM, 10 µM) and cells were incubated overnight. AREG ELISA (R&D, Minneapolis, MN, USA) was performed according to manufacturer’s instructions with supernatants and cell lysates. Cells were lysed in lysis buffer (5 mM Tris-HCl (pH 7.5), 1 mM EGTA, 250 mM saccharose, 1% Triton X-100) supplemented with cOmplete inhibitor cocktail, Roche Applied Science (Penzberg, Germany), and 10 mM 1,10-phenanthroline monohydrate [32]. Supernatants and lysates were analyzed in duplicates. The relative amount of shedding products in the supernatant was calculated in relation to total AREG (supernatant and lysate).

### 2.6. Proliferation Assay

HeLa cells were seeded in 48-well plates. Cells were transfected in the presence or absence of MM (10 µM) and incubated for forty-eight hours. MM was added again after 24 h. Then, cells were harvested using accutase (Merck Millipore, Darmstadt, Germany) and mixed with Trypan blue solution (Sigma Aldrich, St. Louis, MO, USA). The number of viable cells in the solution was determined with Cellometer© Auto 1000 (Nexcelom Bioscience, Lawrence, MA, USA). The assay was performed in quadruplicates.

### 2.7. Automated Western

Western blot was performed as Automated Western, a capillary electrophoresis immunoassay. Cells were lysed in lysis buffer (5 mM Tris-HCl [pH 7.5], 1 mM EGTA, 250 mM saccharose, and 1% Triton X-100) supplemented with cOmplete inhibitor cocktail (Roche Applied Science, Penzberg, Germany) and 10 mM 1,10-phenanthroline monohydrate. Protein concentration was determined by Pierce™ 660nm Protein Assay Reagent (Thermo Fisher Scientific, Waltham, MA, USA). Equal concentrations of protein were loaded and separated by 12–230 kDa Separation Module. Western blot analysis was performed by Automated Western blot analyzer JESS (ProteinSimple, San Jose, CA, USA) with 25 min of separation time at 375 volts, 5 min of blocking, 30 min of primary antibody incubation against the different ANOs (1:10 dilution) or GAPDH (1:30 dilution), and 30 min of secondary antibody incubation according to the manufacturer’s instructions. The results were evaluated with the Compass for SW 5.0 software (ProteinSimple).

### 2.8. Annexin V Staining

HEK293T cells were seeded on μ-Slide 8-well (ibidi, Gräfeling, Germany). Forty-eight hours after transfection and after indicated stimulation periods, cells were immediately incubated with a solution of Annexin V-AF568 (1:50) and Hoechst 33342 (1:1000) in Annexin-Binding-Buffer (ABB: 10 mM HEPES, 140 mM NaCl, and 2.5 mM CaCl_2_, pH 7.4) for 5 min in the dark at room temperature, washed twice with ABB, and fixed for 15 min with 3% paraformaldehyde. After fixation, wells were washed three times with PBS, once with distilled water and covered with mounting medium (ibidi, Gräfeling, Germany). Image acquisition was performed with an Axiovert A1 (Zeiss, Oberkochen, Germany) using a 63×/100× Plan-Apochromat oil immersion objective.

### 2.9. Image Analysis and Statistics

Image analysis was performed with ImageJ version 1.53e (National Institutes of Health, Bethesda, MA, USA). Fluorescence signal above background fluorescence was determined and correlated to the cell number. Four independent experiments were performed. For each experiment one μ-Slide 8-well was prepared and two wells for each group were analyzed, each with five images of different areas. The mean fluorescence per cell number was taken for statistical analysis. Groups were tested by one-way-analysis of variance (ANOVA) and Holm–Sidak multiple comparison post hoc test.

### 2.10. Statistical Analysis

All values are expressed as means ± standard error of the mean (SEM). The standard error values indicate the variation between mean values obtained from at least three independent experiments. Statistics were generated using one-way analysis of variance (one-way ANOVA) and multiple comparison post hoc test as designated. The *p* values < 0.05 were considered statistically significant (either indicated with * or #).

## 3. Results

### 3.1. Ionomycin Stimulation Increases Shedding Activity in Cells Overexpressing ANO4, ANO6 and ANO9

**Figure 1:**
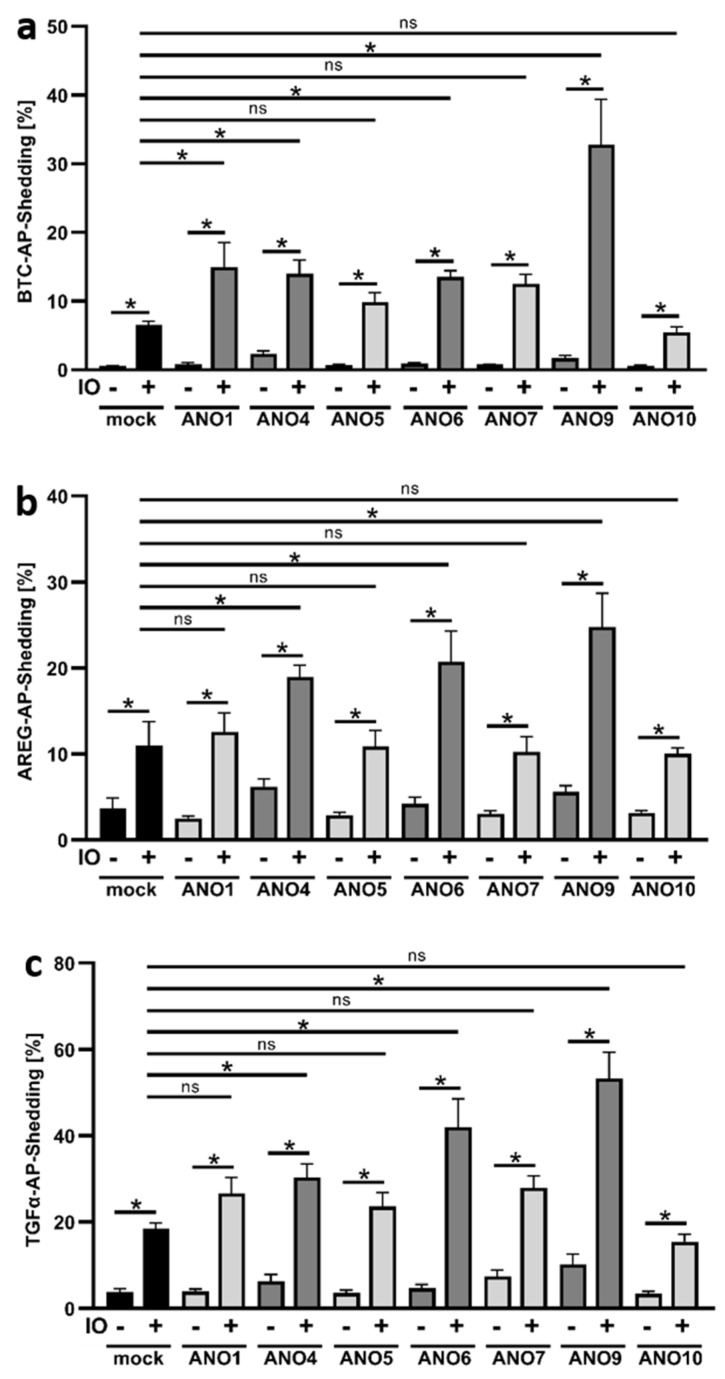
Ionomycin induces increased shedding of BTC, AREG, and TGFα in ANO4-, ANO6-, and ANO9-overexpressing cells. HEK293T cells were co-transfected with an ANO plasmid or mock vector (black columns) and the AP-tagged ADAM10/17 substrates (**a**) betacellulin (BTC), (**b**) amphiregulin (AREG), or (**c**) TGF-alpha (TGFα). Cells were stimulated with ionomycin (IO, 1 µM) for 30 min and analyzed for the release of soluble BTC, AREG, or TGFα via AP-assay. * indicates a significant increase compared to corresponding unstimulated cells or stimulated mock control (dark grey columns), and ns indicates no significant difference (light grey columns) (n = 6; * *p* < 0.05; ±SEM). Data were analyzed by one-way ANOVA and Holm–Sidak multiple comparison post hoc test.

HEK293T cells were co-transfected with respective anoctamin plasmids and alkaline-phosphatase (AP)-tagged ADAM10 (BTC) or ADAM17 substrates (TGFα or AREG). Cells were stimulated 48 h after transfection with ionomycin (IO) for 30 min. AP activity in cell lysates and supernatants was determined and shedding was calculated as percentage of shed substrate compared to total substrate (lysate and supernatant). Ionophore application led to increased shedding of BTC in mock-transfected cells (Figure 1a). As previously observed, IO-stimulated shedding was markedly enhanced in cells co-transfected with ANO6. Significant increases were also observed for ANO1, ANO4, and ANO9. The enhancing effect of ANO9-overexpression was remarkable, far surpassing the originally discovered effect of ANO6. A comparable picture emerged when the release of the preferential ADAM17 substrates AREG (Figure 1b) and TGFα (Figure 1c) was examined. Here, overexpression of ANO4, ANO6, and ANO9 led to significantly enhanced shedding compared to mock-transfected stimulated cells. Again, the most pronounced increases were observed in cells that overexpressed ANO9. Successful ANO transfection was always monitored in parallel by Western blot analyses (Supplementary Figure S1).

### 3.2. PMA Stimulation of ADAM17 Is Independent of Anoctamin Expression

**Figure 2:**
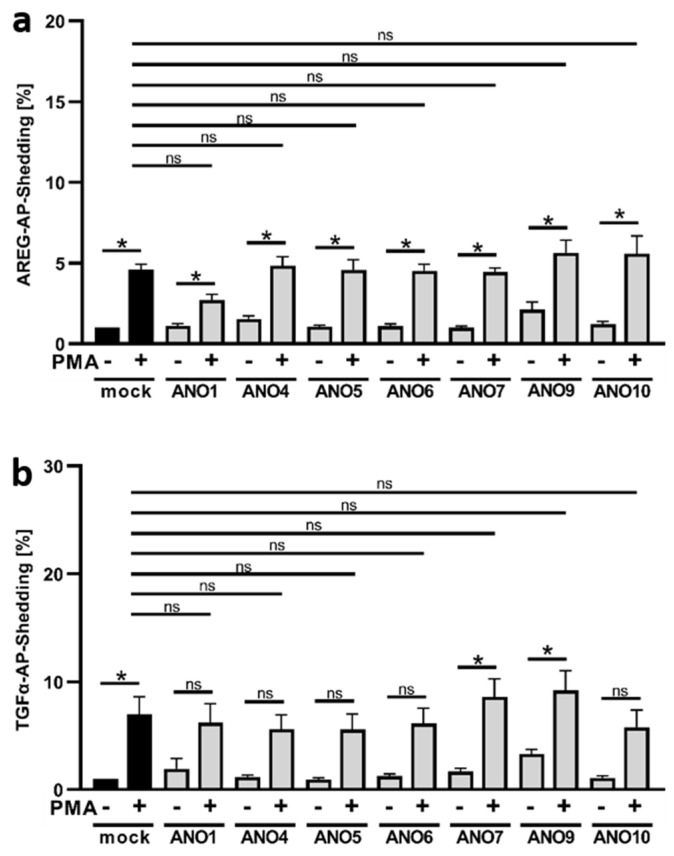
PMA-stimulated release of AREG and TGFα is not affected by overexpression of anoctamins. HEK293T cells were co-transfected with an ANO plasmid or mock vector (black columns) and the AP-tagged ADAM17 substrates (**a**) AREG or (**b**) TGFα. Cells were stimulated with PMA (300 ng/mL) for 30 min and analyzed for the release of soluble AREG or TGFα via AP-assay. * indicates a significant increase compared to corresponding unstimulated cells or stimulated mock control, and ns indicates no significant difference (light grey columns) (n = 3; * *p* < 0.05; ±SEM). Data were analyzed by one-way ANOVA and Holm–Sidak multiple comparison post hoc test.

PMA is a classical ADAM17 stimulus, whereby PKC-activation, rather than intracellular Ca^2+^-elevation, is the cause of PS-exposure that triggers shedding activity. It was, therefore, expected that ANO4- or ANO9-overexpression would not lead to enhanced release of the preferential ADAM17 substrates AREG or TGFα upon PMA stimulation. As shown in Figure 2, this was indeed the case. Enhanced shedding of both AREG (Figure 2a) and TGFα (Figure 2b) evoked by PMA occurred to a perfectly comparable extent in mock- and ANO-transfected cells.

### 3.3. Enhanced TGFα Cleavage in ANO-Overexpressing Cells Involves Other Sheddases Than ADAM17

**Figure 3:**
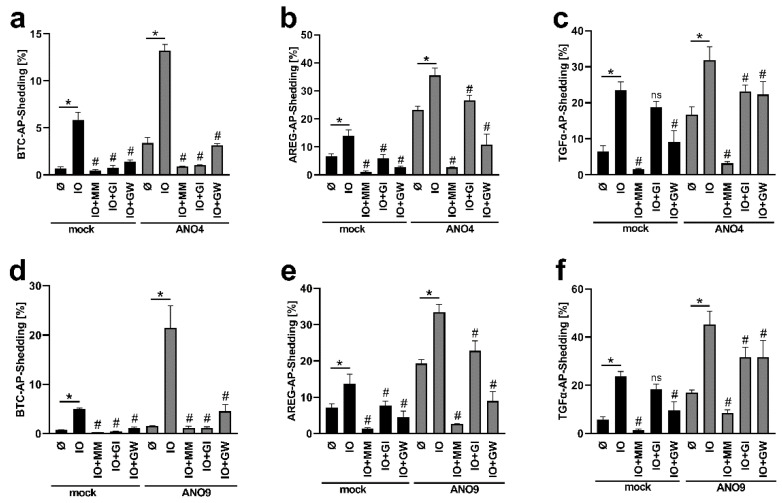
Ionomycin-induced shedding of BTC and AREG in ANO4- and ANO9-overexpressing HEK cells is ADAM10/17-dependent. HEK293T cells were co-transfected with ANO4 (**a**–**c**) or ANO9 (**d**–**f**) (grey columns) or mock vector (black columns) and the AP-tagged ADAM10/17 substrates (**a**,**d**) BTC, (**b**,**e**) AREG, or (**c**,**f**) TGFα. Cells were stimulated with IO (1 µM) for 30 min in the absence or presence of ADAM10 inhibitor GI (3 µM), ADAM10/17 inhibitor GW (3 µM) or broad-spectrum metalloprotease inhibitor marimastat (MM, 10 µM) and analyzed for the release of soluble BTC, AREG or TGFα. * indicates a significant increase compared to unstimulated cells, # indicates a significant decrease compared to corresponding stimulated cells, and ns indicates no significant difference (n = 3; * *p* < 0.05; ±SEM). Data were analyzed by one-way ANOVA and Holm–Sidak multiple comparison post hoc test.

The identity of sheddases involved in substrate cleavage can be probed through the use of preferential proteinase inhibitors. GI254023X (GI) is a preferential inhibitor of ADAM10, and GW280264X (GW) is a dual inhibitor of ADAM10/17. Experiments with cells overexpressing ANO4 are shown in Figure 3a–c. Ionomycin-stimulated shedding in mock-infected controls was within the usual scope (Figure 3a–c, black columns). Shedding of the ADAM10 substrate BTC is well known to be suppressed by GI [16], while cleavage of the preferential ADAM17 substrates AREG and TGFα can be markedly inhibited by GW and partly by GI [24].

Analyses of cells overexpressing ANO4 generated surprising results (Figure 3a–c, grey columns). Stimulated release of BTC was significantly diminished upon application of the preferential ADAM10 inhibitor GI, the mixed ADAM10/17 inhibitor GW and the broad-spectrum metalloproteinase inhibitor marimastat (MM), which indicated that substrate release continued to be affected by ADAM10 in the ANO-overexpressing cells (Figure 3a). Analogously, ADAM17 continued to be primarily responsible for cleaving AREG (Figure 3b). Thus, shedding of this classical ADAM17 substrate was only slightly reduced by ADAM10-inhibitor GI and markedly inhibited by the dual ADAM10/17 inhibitor GW. As in all experiments, the broad-spectrum metalloproteinase inhibitor marimastat (MM) almost totally abrogated all shedding activity (Figure 3).

A different picture emerged for shedding of TGFα, the second preferential ADAM17-substrate (Figure 3c). Overexpression of ANO4 led to enhanced stimulated shedding, which, however, was no longer markedly reduced by ADAM17-inhibition. The dual ADAM10/17 inhibitor GW had a comparable effect to the sole ADAM10 inhibitor GI. Obviously, cleavage of TGFα was no longer affected by ADAM17. This indicated that membrane changes evoked by stimulation of cells with supranormal scramblase activity had in this case led to a re-allocation of the substrate to its shedding enzyme.

Similar experiments were conducted with cells overexpressing ANO9 and the results fully corroborated the above findings (Figure 3d–f). Enhanced shedding of BTC and AREG continued to be due to the action of ADAM10 and ADAM17, respectively (Figure 3d,e). In contrast, the dual ADAM10/17 inhibitor GW exerted no effect further to that achieved by the preferential ADAM10 inhibitor GI on shedding of TGFα (Figure 3f). Thus, cleavage of this substrate was again clearly affected by one or several marimastat-inhibitable proteinases other than ADAM17.

### 3.4. Overexpression of ANO4 or ANO9 Leads to Enhanced PS Exposure

**Figure 4:**
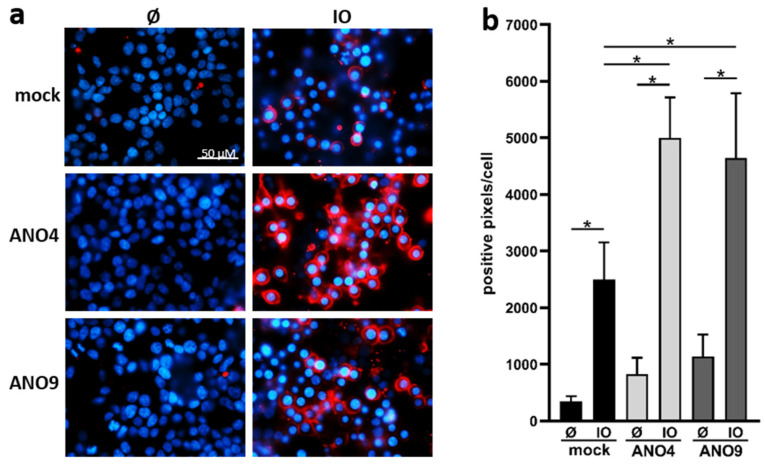
Ionomycin induces increased phosphatidylserine (PS) exposure in ANO4- or ANO9-overexpressing cells. ANO4- or ANO9-transfected HEK293T cells were stimulated with IO (1 µM) for 30 min. After stimulation, cells were stained with Annexin V-AF568 (red). Nuclei were counterstained with Hoechst (blue). Representative images of six independent experiments are shown (**a**). The fluorescence was quantified for statistical analysis (**b**). * indicates a significant increase (n = 6; * *p* < 0.05; ±SEM). Data were analyzed by one-way ANOVA and Holm–Sidak multiple comparison post hoc test.

Overexpression of ANO6 leads to increased Ca^2+^-induced PS-exposure in adherent cells [24]. A similar finding was obtained in cells overexpressing ANO4 and ANO9. Transfected HEK cells were analyzed by staining with fluorescent-labelled Annexin V-AF568 (Figure 4). Increased PS exposure was already observed in ANO4- or ANO9-transfected cells in the absence of stimulation. While these changes did not reach significance, the findings suggest that ANO4 or ANO9 transfection might lead to slightly enhanced constitutive scramblase activity. Higher basal activity of ANO4-expressing cells was reported earlier by Suzuki et al. [21], but the finding has not previously been reported following ANO9-overexpression. After stimulation with ionomycin, PS-exposure was markedly enhanced in transfected cells compared to stimulated mock-transfected cells (Figure 4a,b).

### 3.5. Increased Substrate Cleavage Depends on PS-Interaction

**Figure 5:**
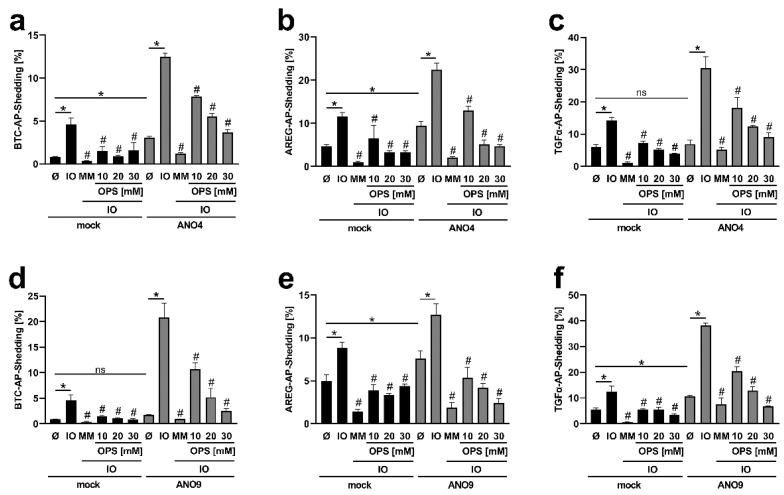
Release of BTC, AREG, and TGFα in ANO4- or ANO9-overexpressing cells depends on surface exposed phosphatidylserine. HEK293T cells were co-transfected with ANO4 (**a**–**c**) or ANO9 (**d**–**f**) vector (grey columns) or mock vector (black columns) and the AP-tagged ADAM10/17 substrates (**a**,**d**) BTC, (**b**,**e**) AREG, or (**c**,**f**) TGFα. Cells were stimulated with IO (1 µM) for 30 min in the absence or presence of inhibitors MM (10 µM) or OPS (10 mM, 20 mM, and 30 mM) and analyzed for the release of soluble BTC, AREG, or TGFα via AP-assay. * indicates a significant increase, # indicates a significant decrease compared to corresponding stimulated cells, and ns indicates no significant difference (n = 3; * *p* < 0.05; ±SEM). Data were analyzed by one-way ANOVA and Holm–Sidak multiple comparison post hoc test.

Competition experiments utilizing the water-soluble head group of PS, ortho-phosphorylserine (OPS), were then conducted to probe whether ANO-dependent enhanced shedding was causally related to surface exposed PS. OPS competes with the interaction of membrane-bound PS with the proteases. The results obtained in ANO4- and ANO9-overexpressing cells are depicted in Figure 5a–f, respectively. The black columns are results obtained in mock-vector controls.

A first scrutiny of the consequences of ANO-overexpression on constitutive shedding (columns 1 and 7) led to an interesting finding. Cells overexpressing both ANO4 and ANO9 appeared to show increased constitutive release of ADAM substrates compared to mock vector controls.

Ionomycin-stimulation then significantly enhanced shedding of all substrates in ANO-overexpressing cells over mock-vector controls. This event could be suppressed in mock as well as in ANO-transfected cells by the simple presence of OPS in the cell medium. It followed that the head group of surface-exposed PS must directly participate in the key event underlying substrate shedding.

**Figure 6:**
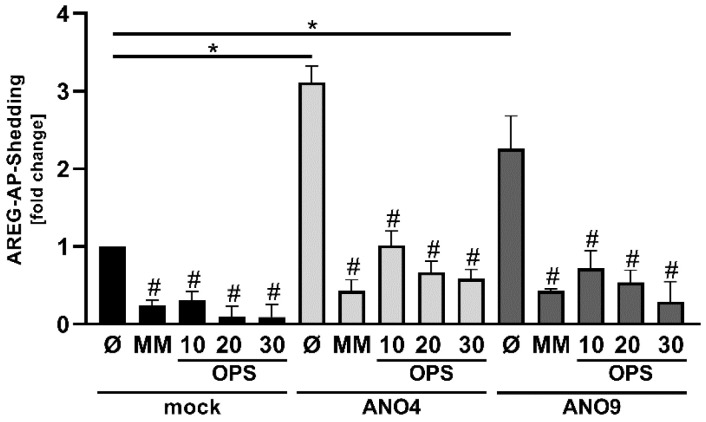
Increased constitutive AREG release in ANO4- or ANO9-transfected cells is inhibited by MM and OPS. HEK293T cells were co-transfected with ANO4 (light grey columns) or ANO9 (dark grey columns) or mock vector (black columns) and the AP-tagged substrate AREG. Forty-eight hours after transfection, cell media were replaced by serum-free media and cells were incubated in the absence or presence of the inhibitors MM (10 µM) or OPS (10 mM, 20 mM, and 30 mM) for 30 min followed by analyses for the release of soluble AREG via AP-Assay. * indicates a significant increase, and # indicates a significant decrease compared to corresponding untreated cells (n = 4; * *p* < 0.05; ±SEM). Data were analyzed by one-way ANOVA and Holm–Sidak multiple comparison post hoc test.

Next, we addressed the issue of constitutive shedding in more detail (Figure 6). The release of soluble AREG was significantly increased upon overexpression of ANO4 as well as ANO9 in the absence of any stimulus. The question was whether this effect could be due to a slight increase of constitutive PS externalization and enhanced PS-dependent ADAM activation. Indeed, the increased shedding was strikingly reduced by application of OPS. The magnitude of reduction was comparable to the effect of marimastat (Figure 6).

### 3.6. Increased Expression of ANO4 and ANO9 Stimulates Cell Proliferation

All the above findings tied in with the notion that PS-translocation to the outer membrane leaflet elicited by ANO4 and ANO9 led to enhanced sheddase function of ADAM10 and ADAM17. The observation that constitutive shedding of AREG appeared increased in cells overexpressing both scramblases opened the way to directly examine their possible biological relevance. AREG is endogenously expressed in epithelial cells and so the spontaneous effects of increased scramblase expression could be easily studied. Enhanced constitutive AREG-release would be expected to directly impact on cell proliferation. Given the relevance of both ADAMs and scramblases to tumor cell function, metastatic cervical cancer (HeLa) cells were used as a model system.

**Figure 7:**
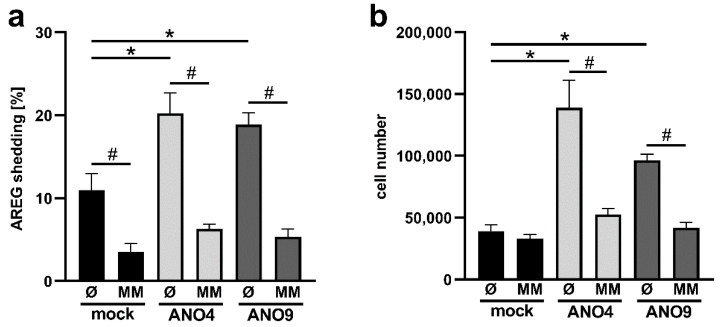
Release of endogenously expressed AREG is constitutively increased upon ANO4- or ANO9-overexpression. HeLa cells were transfected with ANO4 or ANO9 or mock vector. (**a**) Twenty-four hours after transfection cell media were replaced by fresh media. Cells were incubated overnight and then analyzed for the relative amount of shedding products in the supernatant compared to the cell lysate by AREG ELISA. (**b**) Forty-eight hours after transfection, cells numbers were determined. * indicates a significant increase compared to mock-transfected cells, and # indicates a significant decrease compared to corresponding untreated cells (n = 4; *p* < 0.05; ±SEM). Data were analyzed by one-way ANOVA and Holm–Sidak multiple comparison post hoc test.

HeLa cells were transfected with ANO4 or ANO9. After 24 h, medium was changed and cells were incubated in the presence or absence of marimastat. After overnight incubation, cell pellets and supernatants were analyzed by ELISA. As shown in Figure 7a and in confirmation of the previous finding (Figure 4 and Figure 6), overexpression of ANO4 and ANO9 again led to enhanced constitutive shedding of AREG. This effect was totally abrogated in the presence of marimastat.

Cell proliferation can most reliably be assessed by counting viable cells. Compared to mock-transfected cells, the numbers of ANO-transfected cells were significantly increased 48 h after transfection (Figure 7b). This result indicates that both ANO4 and ANO9 promote cell proliferation via enhancement of sheddase function.

## 4. Discussion

We have been studying the possible role of membrane phospholipid asymmetry in regulating the function of cellular sheddases. These studies led to the uncovering of a link between the phospholipid scramblase ANO6 and ADAM10/17 [16,24]. It was found that enhanced scramblase activity leading to increased PS-exposure at the membrane surface directly upregulated proteolytic function of the sheddases. Consequently, we have now proceeded to conduct a systematic analysis to investigate whether this finding might extend to other members of the ANO-family. Affirmative results were obtained for ANO4 and ANO9, and we present evidence for a functional relevance of this ANO-ADAM axis in cell physiology.

HEK cells were co-transfected with ADAM-substrates and various ANOs. ANO1 and ANO10 served as putative negative controls. ANO1 is a calcium-activated chloride channel without scramblase function that controls epithelial secretion, smooth muscle contraction, and sensory signal transduction [33]. Analyses of the ADAM17 substrates TGFα and AREG yielded the expected negative results. Rather surprisingly, however, shedding of the ADAM10 substrate betacellulin was augmented in ANO1-overexpressing cells. It has been reported that ANO1 can positively modulate ANO6-activity [27], and the possibility is given that this might underlie the observed effect.

Anoctamin-10 function has been associated with autosomal recessive cerebellar ataxia [34]. Its scramblase activity seems to be restricted to the endoplasmatic reticulum [35]. An influence on ADAM sheddase activity thus appeared highly improbable and indeed was not observed.

In contrast, plasma membrane localization and PS scramblase activity have been described for ANO4, ANO5, ANO7 and ANO9 [19,20,21].

ANO5 is involved in skeletomuscular function; its mutations cause gnathodiaphyseal dysplasia, an autosomal dominant inherited bone disorder [36,37]. While Suzuki et al. found that transient transfection of cells with ANO5 did not enhance phospholipid scrambling [21], other data suggested the contrary [38,39,40]. Whitlock et al. reported that overexpression of ANO5 led to cell surface localization of ANO5 and to Ca^2+^-dependent phospholipid scrambling in HEK cells [41]. While we confirmed cell surface localization, we observed only slight insignificant increases in PS externalization (data not shown). Our results may differ from the findings of Whitlock for various reasons. Like Suzuki et al., we used a transient expression system while Whitlock et al. used a codon-optimized ANO5 in a stably transfected cell line. The lack of significantly increased PS exposure in our assay accorded with the lack of significantly increased ADAM sheddase activity. The same observations apply to ANO7, a potential scramblase that is upregulated in prostate cancer [42,43,44]. In our hands, overexpression of ANO7 led neither to significantly enhanced PS externalization (data not shown) nor to significant enhancement of ADAM substrate release.

In contrast, the results for ANO4 and ANO9 were unambiguous. Overexpression led to significantly increased PS exposure upon calcium stimulation and significantly increased release of all three ADAM substrates analyzed. In contrast, ANO4- or ANO9-overexpression had no effect on the enhancement of ADAM-function when phorbol ester was employed as stimulus. This underscores the calcium dependency of the ANO-related effects. Inhibition experiments utilizing OPS, the soluble phospholipid head group of PS, provided direct evidence for a role of surface-exposed PS in relaying the ANO-effects to the ADAMs. OPS competes for interaction of the protease with the membrane-exposed phospholipid and was found to dose-dependently diminish shedding of all substrates.

The identity of involved sheddases was probed through the use of the two metalloprotease inhibitors GI (relatively selective for ADAM10) and GW (dual inhibitor of ADAM10 and ADAM17) [30,31]. It is known that TGFα is normally cleaved mainly by ADAM17, but that cleavage can be undertaken by other ADAMs under special circumstances [45,46]. Here, overexpression of ANO4 and ANO9 minimized the inhibitory capacity of GW, indicating that other sheddases had replaced ADAM17. A similar finding had previously been observed upon overexpression of ANO6 [24]. We are speculating that the nanodomain localization of sheddase, substrate, and scramblase will ultimately determine substrate access and substrate cleavage by different ADAMs [47,48].

This would also potentially apply to constitutive sheddase activity. Constitutive scramblase activity independent of elevated calcium has previously been reported for ANO4 [21]. In our study, increased Annexin V-positivity was observed in cells overexpressing both ANO4 and ANO9. Significantly, this was associated with enhanced constitutive release of AREG, but not of TGFα. The increases were again reduced in the presence of OPS, demonstrating PS-dependency also for constitutive substrate release.

The above findings had been made in HEK-cells co-transfected with ADAM-ligands and ANOs. The observation that ANO4 and ANO9 constitutively augmented shedding of the growth factor AREG prompted us to conduct experiments with HeLa cells. These metastatic cervical cancer cells are a useful model to analyze cancer cell function and endogenously express this ADAM-ligand. Overexpression of both ANO4, as well as ANO9, in these cells indeed led to strikingly enhanced constitutive release of AREG, in the absence of any stimulation. Release of EGFR ligands such as AREG can induce cell proliferation migration and/or cell survival via EGFR signaling pathways [49]. Microscopical examination immediately indicated an influence on cell proliferation, and straightforward determinations of viable cell numbers after transfection revealed a truly striking effect of ANO4 and ANO9 on cell proliferation.

The possible functional consequences of these findings are apparent.

Little is known on the biological significance of both scramblases. ANO4 is primarily expressed in tissue with secretory activity and might play a role in aldosterone secretion [50,51]. Moreover, a role in the Central Nervous Systemhas been postulated as well as an association with various neuronal disorders [52,53,54,55]. In addition, Single-Nucleotide Polymorphisms in the ANO4 gene are associated with breast cancer [56,57].

ANO9 expression has been linked to several malignancies [58,59,60]. ANO9 downregulation in colorectal cancer reportedly plays a role in tumorigenesis and cancer progression. However, an opposite function was described for esophageal squamous cell carcinoma. Katsurahara et al. demonstrated that depletion of ANO9 resulted in reduced cell proliferation, invasion, and migration. Clinically, patients with high ANO9 expression exhibited significantly worse survival [61].

Moreover, ANO9 has been suggested as clinically useful prognostic marker for pancreatic cancer and a potential therapeutic target. The scramblase is overexpressed in pancreatic cancer cells, and high ANO9 expression is a poor prognostic factor in patients with pancreatic cancer. Data by Jun et al. suggest that ANO9 promotes tumorigenesis via increased EGFR expression [59]. If overexpression of ANO9 in pancreatic cancer cells would additionally correlate with increased ADAM sheddase activity and increased release of EGFR ligands, accelerated tumor progression and metastasis could be the detrimental consequences.

Now, first evidence is provided that ANO4 and ANO9 upregulate ADAM10 and ADAM17 function upon calcium influx stimulation, but also on a constitutive level. In particular, increased release of EGFR ligands may indicate a possible participation of the scramblase-ADAM network in regulating cellular functions. Both ADAMs, as well as the anoctamins, have been shown to play a role in the development and progression of multiple cancer types. ADAM and anoctamin inhibitors are discussed for cancer therapy; thus, the functional relevance of scramblases in the general context of ADAM activation needs to be followed up in future investigations.

## Data Availability

Not applicable.

## Supplementary Materials

The following supporting information can be downloaded at: www.mdpi.com/article/10.3390/membranes12020123/s1, Figure S1: Representative Western blots of ANO transfected HEK293T cells; Figure S2: Original uncropped data of Figure S1.
