# Supplementary material for: Influence of Anoctamin-4 and -9 on ADAM10 and ADAM17 Sheddase Function
Source: Membranes (Basel). 2022 Jan 20;12(2):123. doi: 10.3390/membranes12020123 (PMC8879676; doi:10.3390/membranes12020123)
Supplement: Supplementary file 1 [file membranes-12-00123-s001.zip › membranes-1536877-supplementary.pdf]

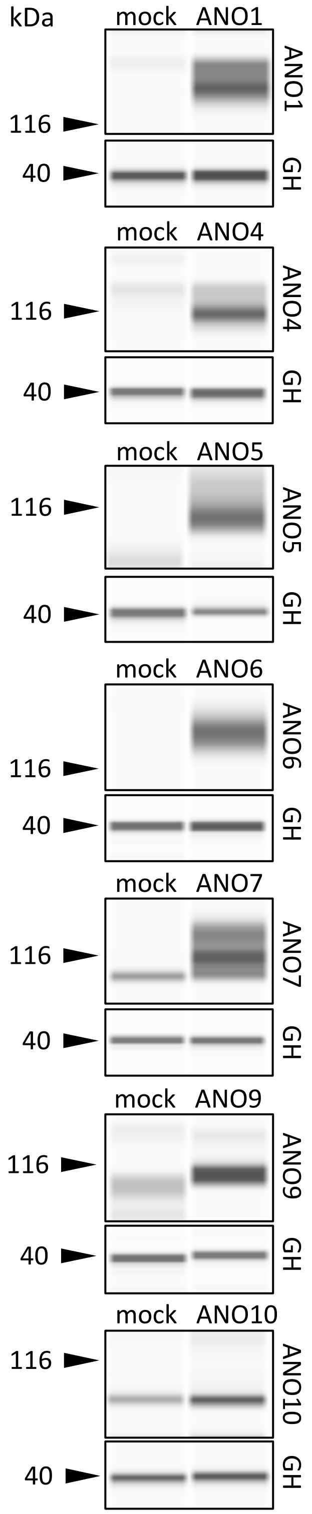

Supplemantary Figure S1: Representative Western blots of ANO transfected HEK293T cells. In parallel to the AP-assays, HEK cells were always analysed for successful ANO transfection by automated Western (see Materials and Methods). The labelling on top indicates the respective transfection (mock or ANO). The side labelling indicates the respective antibodies used. Parallel staining of the housekeeping protein GAPDH (GH) was used as loading control.

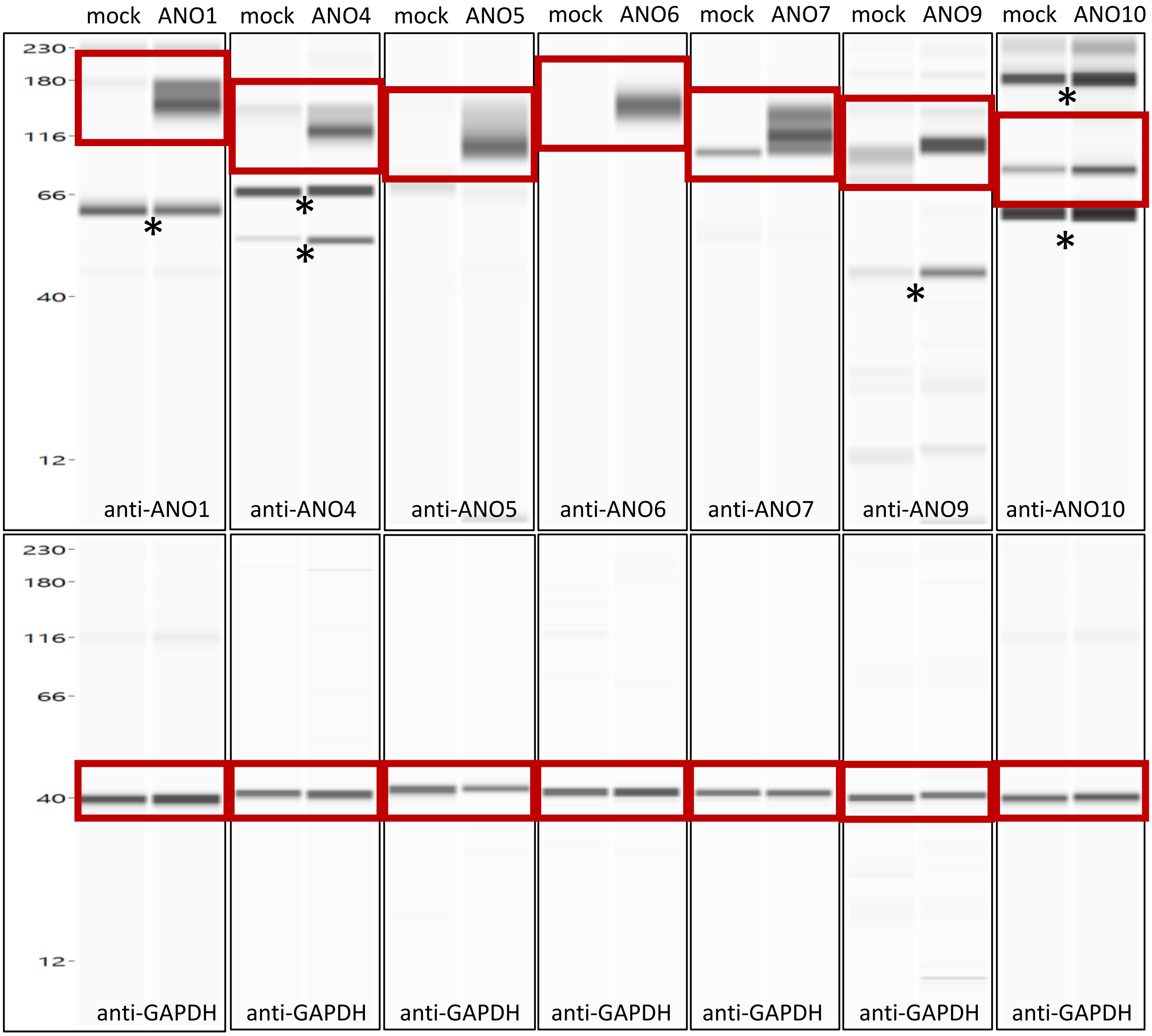

Supplementary Figure S2: Original uncropped data of Figure S1. Most ANO proteins exist as non-glycosylated and glycosylated proteins, therefore, typically two bands are detected in most cell types.
* bands of unknown origin.
